# Industrial Training and After-Care

**Published:** 1912-05-04

**Authors:** 


### THE DEFECTIVE AND DEPENDENT.*

### VIII.—Industrial Training and After-Care.

In preceding papers reference has been made more especially to the scholastic education of the afflicted classes, and in the present we propose to deal in further detail with their industrial training and with the after-care which is so essential after school-age to utilise the results of training as a means of livelihood in order to rescue those handicapped by physical and mental infirmities from a state of absolute dependence.

### The Blind.

In connection with the education of the blind we briefly referred to some of the handicrafts taught them. We may now add that the industrial training of those who have passed through the special school course has been facilitated by the recognition by the Board of Education of trade schools for blind adolescents as a grant-earning form of technical instruction, and a committee designated "The Workshops for the Blind of London Federation Board" has recently been formed to co-ordinate the various efforts to promote the employment of the blind in the Metropolitan area. After-care committees have been organised to aid trained pupils to secure suitable situations, or, when necessary, to obtain funds for their apprenticeship from such charities as "Gardner's Trust" or associations for the benefit of the blind, which exist in considerable number throughout the kingdom. For those brought up in residential institutions, attached workshops are in most cases available, which are run upon a commercial basis, a staple trade being often adopted as may be found most profitable in a particular locality. Thus in the blind asylums of Edinburgh and Glasgow the manufacture of bedding takes the lead, whilst at the Birmingham, Liverpool, and Belfast institutions brush and broom making is considered the best paying industry.

At the Leicester, Leeds, Bradford, and Nottingham institutions basket-making is extensively practised; in others preference is given to the manufacture of mats, matting, and fancy rugs, the last being a special feature at the School for the Indigent Blind at Leatherhead. Skilled handicrafts are thus acquired by the more intelligent blind, but for those less expert home occupations of a simple kind, such as chair-caning, sack-making, firewood cutting, etc., are available, with hand and machine knitting for women. At the other end of the scale there is provision for higher education and professional training, and the Royal Normal College for the Blind at Upper Norwood prepares its students to qualify as certificated teachers of the blind, as organists and teachers of music, as piano-tuners and as typists. At Henshaw's Blind Asylum, Manchester, a specialty is made of massage as an occupation for which the blind are well qualified owing to their cultured tactile sensibility. Notwithstanding facilities for industrial training, however, many obstacles exist in the way of securing permanent remunerative employment, especially in the case of those entirely blind, and efficient after-care is indispensable to ensure economical results.

Of late years there has been a considerable extension of manual training for junior deaf pupils and of industrial training for the seniors. Kindergarten departments have been established in connection with the schools, and industrial training departments have been formed for adolescents. The London County Council have two senior schools for the deaf, one at Anerley for ninety boys and another at Wandsworth Common for sixty girls, in which industrial training is given irrespective of language attainments. At the Manchester Royal Schools for the Deaf there is a special industrial training department for thirty youths, who are taught tailoring and bootmaking. After-care reports give an encouraging view as to the future of the trained deaf of normal mental capacity. Thus the Preston Institution reports forty-six male pupils as earning their living out of sixty-seven who had passed through training, and in addition eleven others capable of doing so, wholly or in part; twenty-eight out of fifty-eight girls trained in the institution are earning good wages, four are apprenticed, four suitably employed at home, two partially earning their living, two capable girls out of work, and six living at home with their families in comfortable circumstances. The general level of employment for the deaf, as also for the blind, seems improving since the provision for education for those two classes has become general, and the blind beggar and the deaf and dumb tramp are happily becoming markedly diminished in number.

### The Mentally Defective.

The essential importance of manual instruction in this class, who are only capable of education by concrete methods and learn more through the fingers than by direct appeal to the brain, has already been pointed out. It is also obvious that even those who have been trained to useful mechanical work as a rule show little power of independent initiative, and if their attainments are to be utilised for remunerative employment, some form of after-care is indispensable. As Sir George Newman says in his Report to the Board of Education for 1910: "It is only possible to form a safe opinion of the value of the training of the feeble-minded where an after-care committee exists." Reports of after-care committees in the various centres of special instruction in England show a somewhat surprising discrepancy in their statistics of results, depending probably upon varying standards of intelligence considered as qualifying for such instruction and also as regards the arrangements made for indus-

* Previous articles appeared on Oct. 7 and 21, Nov. 18, Dec. 2, Jan. 6, Feb. 10 and 17, March 2, 16, and 23.

trial as distinguished from merely scholastic training. Thus, from statistics collected from sixteen centres cited at the After-care Conference of 1911 of the National Association for the Feeble-minded, it would appear that only 30.4 per cent. of the cases who have passed through special schools are doing serviceable or remunerative work, the London centres included showing 63.9 per cent., while those at Birmingham show only 22.7 per cent., and at Liverpool only 10.5. In London, where special industrial schools have been organised for the senior boys, it is stated in the Report for 1909 of the Chief Medical Officer to the Board of Education that of 457 boys who have passed through these schools during the last six years 208 (or 45.7 per cent.) are in good and promising work, receiving wages from 8s. to 20s. per week (or even more), in addition to 20 per cent. who are at present employed in precarious or ill-paid occupations. Some 34 per cent. are classed as unsatisfactory, requiring permanent care in their own interests and that of the community. From the standpoint of eugenics, indeed, a much larger proportion of the feeble-minded class generally require to be segregated in order to diminish the propagation of the unfit in future generations.

